# Glial reactivity in a mouse model of beta-amyloid deposition assessed by PET imaging of P2X7 receptor and TSPO using [^11^C]SMW139 and [^18^F]F-DPA

**DOI:** 10.1186/s13550-024-01085-7

**Published:** 2024-03-06

**Authors:** Obada M. Alzghool, Richard Aarnio, Jatta S. Helin, Saara Wahlroos, Thomas Keller, Markus Matilainen, Junel Solis, Jonathan J. Danon, Michael Kassiou, Anniina Snellman, Olof Solin, Juha O. Rinne, Merja Haaparanta-Solin

**Affiliations:** 1 PET Preclinical Imaging Laboratory, Turku PET Centre, University of Turku, Tykistökatu 6 A, 20520 Turku, Finland; 2 Medicity Research Laboratory, University of Turku, Tykistökatu 6 A, 20520 Turku, Finland; 3 Drug Research Doctoral Programme, University of Turku, Turku, Finland; 4 Turku University Hospital, Turku PET Centre, Kiinamyllynkatu 4-8, 20520 Turku, Finland; 5 Radiopharmaceutical Chemistry Laboratory, Turku PET Centre, University of Turku, Kiinamyllynkatu 4-8, 20520 Turku, Finland; 6 Turku BioImaging, Åbo Akademi University and University of Turku, Turku, Finland; 7 School of Chemistry, The University of Sydney, Sydney, NSW 2006 Australia; 8 Department of Chemistry, University of Turku, Henrikinkatu 2, 20500 Turku, Finland; 9 Accelerator Laboratory, Turku PET Centre, Åbo Akademi University, Kiinamyllynkatu 4-8, 20520 Turku, Finland; 10 Department of Neurology, Turku University Hospital, Kiinamyllynkatu 4-8, 20520 Turku, Finland

**Keywords:** Positron emission tomography, [^11^C]SMW139, [^18^F]F-DPA, Alzheimer’s disease, APP/PS1-21, Microglia, P2X7, TSPO, P2Y12

## Abstract

**Background:**

P2X7 receptor has emerged as a potentially superior PET imaging marker to TSPO, the gold standard for imaging glial reactivity. [^11^C]SMW139 is the most recently developed radiotracer to image P2X7 receptor. The aim of this study was to image reactive glia in the APP/PS1-21 transgenic (TG) mouse model of Aβ deposition longitudinally using [^11^C]SMW139 targeting P2X7 receptor and to compare tracer uptake to that of [^18^F]F-DPA targeting TSPO at the final imaging time point. TG and wild type (WT) mice underwent longitudinal in vivo PET imaging using [^11^C]SMW139 at 5, 8, 11, and 14 months, followed by [^18^F]F-DPA PET scan only at 14 months. In vivo imaging results were verified by ex vivo brain autoradiography, immunohistochemical staining, and analysis of [^11^C]SMW139 unmetabolized fraction in TG and WT mice.

**Results:**

Longitudinal change in [^11^C]SMW139 standardized uptake values (SUVs) showed no statistically significant increase in the neocortex and hippocampus of TG or WT mice, which was consistent with findings from ex vivo brain autoradiography. Significantly higher [^18^F]F-DPA SUVs were observed in brain regions of TG compared to WT mice. Quantified P2X7-positive staining in the cortex and thalamus of TG mice showed a minor increase in receptor expression with ageing, while TSPO-positive staining in the same regions showed a more robust increase in expression in TG mice as they aged. [^11^C]SMW139 was rapidly metabolized in mice, with 33% of unmetabolized fraction in plasma and 29% in brain homogenates 30 min after injection.

**Conclusions:**

[^11^C]SMW139, which has a lower affinity for the rodent P2X7 receptor than the human version of the receptor, was unable to image the low expression of P2X7 receptor in the APP/PS1-21 mouse model. Additionally, the rapid metabolism of [^11^C]SMW139 in mice and the presence of several brain-penetrating radiometabolites significantly impacted the analysis of in vivo PET signal of the tracer. Finally, [^18^F]F-DPA targeting TSPO was more suitable for imaging reactive glia and neuroinflammatory processes in the APP/PS1-21 mouse model, based on the findings presented in this study and previous studies with this mouse model.

**Supplementary Information:**

The online version contains supplementary material available at 10.1186/s13550-024-01085-7.

## Background

Sustained glial reactivity to pathological changes underlies neuroinflammation in Alzheimer’s disease (AD) [1] and other neurodegenerative disorders [2]. Glial reactivity in the brain could be beneficial by degrading beta-amyloid (Aβ) plaques, or detrimental by secreting cytokines and other promoters of neuroinflammation [3]. In response to disease, microglia undergo distinct changes in shape, count, function, and upregulation of surface bioactive molecules expression [1]. Purinergic 2 type X receptor subtype 7 (P2X7) is a key surface receptor in microglia, the expression of which is upregulated when the cells are in a reactive state [4]. P2X7 receptor is an ATP-gated ion channel that is widely distributed in the brain and expressed abundantly in microglia, but also in neurons and other glial cells, such as astrocytes and oligodendrocytes [5]. Among the physiological roles of P2X7 receptor is regulating neurotransmitters release [6]. P2X7 receptor also has several immune response functions in reactive microglia; it is implicated in the release of pro-inflammatory cytokines, production of reactive oxygen and nitrogen species, caspase activation, and the induction of apoptosis, collectively fueling neuroinflammation [4].

In the context of AD, P2X7 receptor expression has been observed to co-localize with microglia surrounding Aβ plaques in the brains of AD patients and rodent models of AD [7–9]. It is a well-established fact that Aβ causes microglial reactivity in AD [10–12]. Microglia react to Aβ plaques by releasing various pro-inflammatory cytokines, and upregulating P2X7 receptor expression, a response that potentially contributes to the inflammatory environment around Aβ plaques [13, 14]. Interestingly, P2X7 receptor seems to play a fundamental role in microglial response to Aβ plaques, as P2X7-deficient microglia were not reactive to Aβ plaques in mice [14]. The upregulated expression of P2X7 receptor in AD has also been associated with reactive microglia inflammatory reaction to neurodegeneration and tissue damage [13, 15]. The purinergic 2 type Y receptor subtype 12 (P2Y12) is another key receptor in microglia, the expression of which is downregulated in the brain of patients with AD and tauopathies and mouse models of tau pathology [16]. Accordingly, P2X7 and P2Y12 receptors have attracted a lot of interest as positron emission tomography (PET) imaging markers of reactive microglia and neuroinflammation.

P2X7 receptor emerged as a potentially superior imaging marker to translocator protein-18 kDa (TSPO), which remains the gold standard for imaging glial reactivity despite its drawbacks, including the low brain uptake and high non-specific binding of TSPO-targeting radiotracers, and TSPO polymorphism effect on the binding affinity between subjects [17, 18]. A number of potent and selective P2X7 receptor antagonist ligands have already been developed as PET radiotracers [19]. However, only [^11^C]JNJ-54173717 [20], [^18^F]JNJ-64413739 [21] and the most recently developed radiotracer targeting P2X7 receptor, [^11^C]SMW139 [22], advanced from in vitro and preclinical development to clinical evaluation. [^11^C]SMW139 preliminary assessment in a small cohort of MS patients demonstrated slightly higher brain uptake compared to healthy controls [23]. Preclinical studies showed that [^11^C]SMW139 could detect increased expression of human and endogenous rat P2X7 receptor in rats [22, 24], despite the lower binding affinity of [^11^C]SMW139 to rodent than human P2X7 receptor [24, 25]. However, [^11^C]SMW139 binding on post-mortem brain sections did not differentiate AD patients from healthy subjects [22], which raised concerns on [^11^C]SMW139 applicability to image P2X7 receptor in AD.

In our study, we used the APP/PS1-21 transgenic (TG) mouse model of Aβ deposition, a reliable model to validate radiotracers in preclinical settings, which has been used in the development of novel PET radiotracers targeting reactive glia [26, 27] and misfolded proteins, such as tau [28]. In this model, early onset Aβ plaques are associated with simultaneous and robust neuroinflammation, represented by the presence of increased reactive microglia around the Aβ plaques [29]. The aim of the present study was to image reactive glia in the APP/PS1-21 TG mouse model of Aβ deposition longitudinally with [^11^C]SMW139 targeting P2X7 receptor and compare tracer uptake in the same mice to that of [^18^F]F-DPA targeting TSPO at the final imaging time point. In previous studies, [^18^F]F-DPA targeting TSPO in the APP/PS1-21 mouse model has shown the ability to image reactive glia, differentiate transgenic from age-matched wild type mice, and superior brain uptake to the TSPO PET tracers [^18^F]DPA-714 and [^11^C] PBR28 [30, 31]. This study contributes to the development of [^11^C]SMW139 and [^18^F]F-DPA for imaging reactive glia in the APP/PS1-21 mouse model.

## Methods

### Radiotracers

The radiotracers [^11^C]SMW139 (2-chloro-5-[^11^C]methoxy-*N*-((3,5,7-trifluoroadamantan-1-yl)methyl)benzamide) and [^18^F]F-DPA (*N*,*N*-diethyl-2-(2-(4-([^18^F]fluoro)phenyl)-5,7-dimethylpyrazolo[1,5-a] pyrimidin-3-yl)acetamide) were produced in the radiopharmaceutical laboratory at Turku PET Centre as described previously [32, 33]. For the 32 batches of [^11^C]SMW139, the molar activity was 33.0 (21.0) GBq/µmol at the time of injection, radiochemical purity 98.3% (0.5%), and radiotracer shelf-life 1 h. For the three batches of [^18^F]F-DPA, the molar activity was 3.2 (0.6) GBq/µmol at the time of injection, radiochemical purity > 99%, and radiotracer shelf-life at least 4 h.

### Animals

The APP/PS1-21 TG mice and their wild type (WT) littermates were used in the longitudinal in vivo PET imaging. In the ex vivo studies, additional C57BL/6 J WT mice were used with the PET-imaged mice (after imaging was completed). One to four mice were housed in individually ventilated cages with ad libitum access to soy-free chow (RM3 (E) Soya Free, Special Diets Service, Essex, UK) and tap water under the following housing conditions: temperature 21 °C (3 °C), humidity 55% (15%), light cycle 12 h (7:00 to 19:00 light), and aspen wood bedding.

The APP/PS1-21 mice were originally provided by KÖESLER (Rottenburg, Germany). The colony was bred and maintained with C57BL/6Cn mice according to the guidelines of the International Council of Laboratory Animal Science (ICLAS) in the Central Animal Laboratory of the University of Turku. Animal studies were performed in accordance with the European Ethics Committee (decree 86/609/CEE), and adhered to the ARRIVE guidelines, except randomization or blinding [34]. In addition, animal studies adhered to the 3Rs principle (Replacement, Reduction, and Refinement) by employing a longitudinal in vivo PET imaging design, and utilizing tissue samples from the same mouse efficiently for all ex vivo studies. The Animal Experiment Board of the Province of Southern Finland (ESAVI/4660/04.10.07/2016) granted ethical approval of this study.

### In vivo PET imaging

**Fig. 1:**
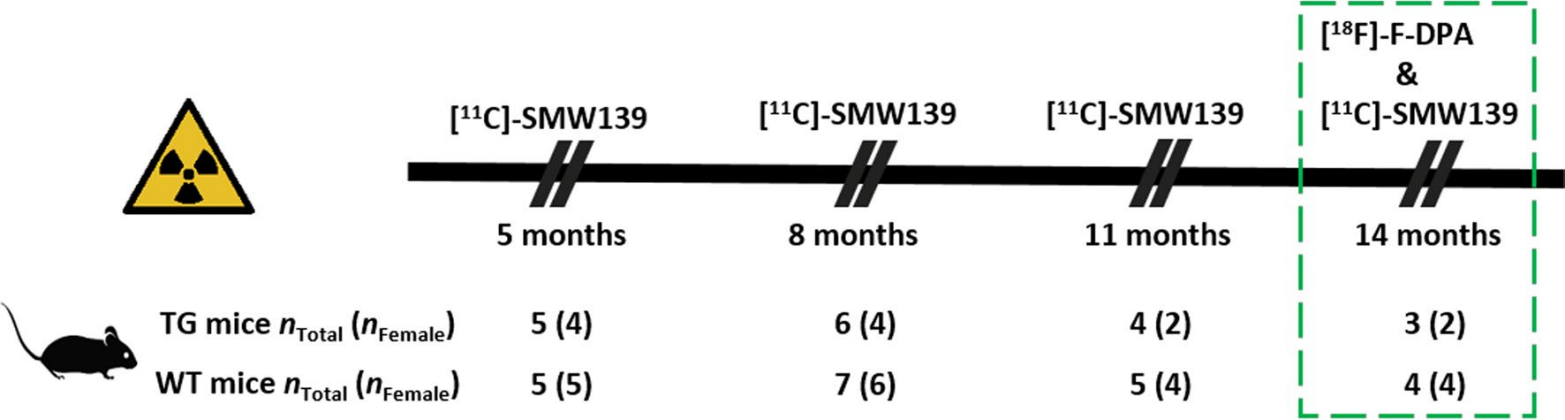
In vivo PET imaging study design and mice counts at each imaging time point. The green box illustrates that at 14 months, mice were imaged with both radiotracers [^11^C]SMW139 and [^18^F]F-DPA. *TG* transgenic, *WT* wild type

The longitudinal in vivo PET imaging using [^11^C]SMW139 with a 13-mouse cohort (TG *n* = 6, including 4 females; WT *n* = 7, including 6 females) consisted of a baseline scan at the age of 5 months and three follow-up scans at 8, 11, and 14 months. In addition, mice were imaged with [^18^F]F-DPA after the final [^11^C]SMW139 scan at 14 months, and both tracers brain uptake was compared only at this time point. At 5 months, PET scans were not obtained from 1 TG and 2 WT mice due to unsuccessful radiotracer injections. At 8 months, PET scans were successfully obtained from all TG and WT mice. At 11 months, 2 TG and 2 WT mice had to be sacrificed due to poor health conditions, and they were not scanned. Similarly, at 14 months, 1 TG and 1 WT mice needed to be sacrificed and were not scanned. The study design and final mice count at each time point are presented in Fig. 1. The mice were sacrificed after the final PET scan by cardiac puncture under deep isoflurane anesthesia and received cardiac perfusion with saline, then used in ex vivo brain autoradiography, immunohistochemical staining and radiometabolite analysis.

The same PET imaging protocol was used with [^11^C]SMW139 and [^18^F]F-DPA; the mice were imaged in pairs using the Inveon Multimodality PET/CT scanner (12.7 cm axial and 10 cm transaxial field of view, PET camera spatial resolution 1.4 mm, energy window 350–650 keV; Siemens Medical Solutions, Knoxville, TN). Mice were anesthetized with inhaled isoflurane (300 mL/min 3.5% isoflurane/O_2_ for induction and 2% for maintenance), cannulated in the lateral tail vein and moved to the scanner, where a CT scan was first performed for attenuation correction and anatomical reference. The mice received an intravenous bolus injection of the radiotracer ([^11^C]SMW139: injected activity 9.5 (0.5) MBq, injected mass 6.06 (5.61) µg/kg; [^18^F]F-DPA: injected activity 6.7 (0.5) MBq, injected mass 22.5 (4.32) µg/kg; injected volume ≤ 200 μL) and underwent a dynamic 60 min (min) PET scan. During the scans, the mice laid prone on a heating pad and their eyes were protected from dryness with an ophthalmic gel (Oftagel, Santen Oy, Tampere, Finland).

PET data was converted from 3D list mode to 2D sinograms by a Fourier rebinning algorithm, then reconstructed with a 2D-filtered back-projection algorithm into an image with a voxel size of 0.78 × 0.78 × 0.80 mm, or approximately 0.5 mm^3^. Image data were divided into 49-time frames (30 × 10 s, 15 × 60 s, 4 × 300 s) and decay-corrected to the injection time. PET images were analyzed using Inveon Research Workplace analysis software v. 4.2 (Siemens Medical Solutions). Rigid co-registration to a 3D magnetic resonance imaging (MRI) mouse brain template (MRM NAt Mouse Brain Database, McKnight Brain Institute) was used as an anatomical reference for the CT and corresponding dynamic PET images of each mouse. The PET image and MRI template were positioned by translation (moving on X, Y, Z-axes)/angle rotation within the CT image. Brain uptake of the radiotracers was quantified in the hippocampus as a representative non-cortical region, and neocortex on the co-registered CT image and MRI template in the absence of the PET image (i.e., not guided by observed radioactivity in the brain to avoid potential bias). The size of each volume of interest (VOI) template was adjusted according to the size of the brain, so that almost identical volumes were analyzed. The averaged standardized uptake values (SUVs) for [^11^C]SMW139 were calculated in the 3–15 min time frame as already 30 min after the radiotracer injection, at least 46% of brain radioactivity originated from the radiometabolites (more details in the discussion). Averaged SUVs for [^18^F]F-DPA were calculated in the 25–50 min time frame. Regional time-activity curves were plotted for the completely dynamic scans.

### Ex vivo brain autoradiography

30 mice (TG *n* = 13, including 6 female; WT *n* = 17, including 8 female) at 5, 8–10, 12 and 14 months (including the longitudinally imaged mice) (Table 1) were used to investigate [^11^C]SMW139 binding on mouse brain cryosections using ex vivo autoradiography. Mice were sacrificed 10 min after [^11^C]SMW139 injection (injected dose 10.0 (0.7) MBq), with cardiac puncture under deep isoflurane anesthesia and received cardiac perfusion with saline. Mice brains were then dissected, frozen and sliced as described previously [26]. From each mouse, cryosections were sliced at the level of cortex and thalamus as a representative non-cortical region. For both brain regions, ^11^C-radioactivity intensity expressed as background-erased photo-stimulated luminescence per pixel (PSL/pixel—Bkg) was obtained by manually drawing a region of interest on the cortex and thalamus from the mouse sections. Regional uptake of [^11^C]SMW139 was then calculated as SUV using the PSL/pixel-Bkg of cortex and thalamus and the pre-measured cortical percentage injected activity per gram value. Autoradiography images were analyzed using Aida Image Analyzer v. 4.5 (Raytest Isotopenmessgeräte GmbH, Straubenhardt, Germany).

**Table 1:** Mice used in ex vivo studies

	Age (mo)	Genotype	N_GENDER_		N_TOTAL_
			F	M	
Ex vivo brain autoradiography	5	TG	3	1	4
		WT	2	2	4
	8–10	TG	3	2	5
		WT	2	5	7
	12	TG	–	2	2
		WT	–	1	1
	14	TG	1	1	2
		WT	3	2	5
P2X7 immunohistochemical staining	5	TG	1	–	1
		WT	–	1	1
	8–10	TG	1	1	2
		WT	–	2	2
	12	TG	–	2	2
		WT	–	2	2
	14	TG	1	–	1
		WT	1	–	1
	15	TG	1	–	1
		WT	–	–	–
TSPO & P2Y12 immunohistochemical staining	5	TG	2	1	3
		WT	1	2	3
	8–10	TG	2	1	3
		WT	1	2	3
	12	TG	–	3	3
		WT	–	2	2
	14	TG	2	1	3
		WT	4	–	4
	15	TG	1	2	3
		WT	–	–	–

	Δt (min)	Genotype	N_GENDER_		N _TOTAL_
			F	M	
Plasma radiometabolites analysis	10	TG	4	9	13
		WT	8	8	16
	30	TG	3	–	3
		WT	5	8	13
	45	TG	3	–	3
		WT	9	2	11
Brain radiometabolites analysis	10	TG	4	2	6
		WT	7	3	10
	30	TG	3	–	3
		WT	5	9	14
	45	TG	3	–	3
		WT	9	1	10

*mo* month, *min* minutes, *F* female, *M* male, *TG* transgenic, *WT* wild type

### Immunohistochemical staining

The expression of P2X7 receptor, TSPO and purinergic 2 type Y receptor subtype 12 (P2Y12) receptor was investigated in TG and WT mice at 5, 8–10, 12, 14, and 15 months (Table 1) utilizing cryosections used in ex vivo brain autoradiography. Immunohistochemical staining was performed using the semi-automated Labvision autostainer (ThermoFisher Scientific). The staining protocols are described in Additional file 1: Table S1. P2X7 receptor staining (*n* = 13, including 7 TG), TSPO and P2Y12 receptor staining (*n* = 27, including 15 TG) were performed in the same TG and WT mice only at 5 and 12 months. Staining were performed in all animals using non-consecutive slides.

The stained brain sections were digitized using Panoramic 250 Flash slide scanners (3DHistech, Budapest, Hungary). Images were examined using Case Viewer v. 2.1 (3DHistech). P2X7 receptor, TSPO and P2Y12 receptor staining were quantified from the digitized brain section images as positive object counts/mm^2^ from two sections/mouse. The size of the quantification region of interest matched that of thalamus (both hemispheres) and cortex (one hemisphere). TSPO and P2Y12 receptor quantification was achieved in QuPath software using the positive cell detection function [35]. P2X7 receptor quantification was achieved using the artificial intelligence object detection algorithm You Only Learn One Representation (YOLOR) [36]. A description of the analytical workflow and image processing source code for the quantification of P2X7 receptor staining are available in Additional file 1. Negative staining was performed to verify the specificity of the P2X7 receptor and TSPO antibodies (Additional file 1: Fig. S1).

### Radiometabolite analysis

To understand the in vivo metabolic profile of [^11^C]SMW139 in mice, and to assist in the imaging data quantification, plasma and brain homogenate samples from TG and WT mice, including the ex vivo brain autoradiography mice cohort (Table 1) were analyzed by thin-layer chromatography or high-performance thin-layer chromatography (Protocols described in Additional file 1) for the fraction of unmetabolized [^11^C]SMW139 (i.e., parent fraction). Cardiac blood (~ 500 μL) and brain tissue samples were collected after 10, 30, or 45 min of [^11^C]SMW139 injection. Mice sacrificed at 10 min were divided into 5, 8–10, and 12–15 months, at 30 min into 3 and 7–10 months, and at 45 min as 7–10 months. Aida Image Analyzer v. 4.5 was used to calculate the percentage of unmetabolized [^11^C]SMW139 from the total ^11^C-radioactivity in the samples.

### Statistical analysis

In all studies, results are reported as mean and standard deviation in parentheses when *n* ≥ 3, or only the mean or individual values when *n* < 3. In the longitudinal in vivo PET imaging study, linear mixed model with compound symmetry covariance structure were used to assess the relationship between [^11^C]SMW139 SUVs in the investigated VOIs at all time points with one within-subject factor (time) and one between-subjects factor (TG and WT group), as well as their interaction term. The interaction term examined whether the groups had different trends of change over time. If the interaction term was deemed not significant, it was dropped from the model. As a post-hoc analysis, differences in least squares means were assessed for all significant factors in order to find the individual differences between groups and time points. The difference in [^11^C]SMW139 and [^18^F]F-DPA SUVs between TG and WT mice in the investigated VOIs at 14 months was evaluated using t-test with Welch’s correction (unpaired, parametric), after datasets were tested for normalization using Shapiro–Wilk's test. All statistical tests were performed as two-sided with the threshold for statistical significance set at 0.05. All analyses were performed using SAS software (version 9.4 for Windows; SAS Institute Inc., Cary, NC, USA).

## Results

### Longitudinal in vivo PET imaging with [^11^C]SMW139 and comparison to [^18^F]F-DPA

**Fig. 2:**
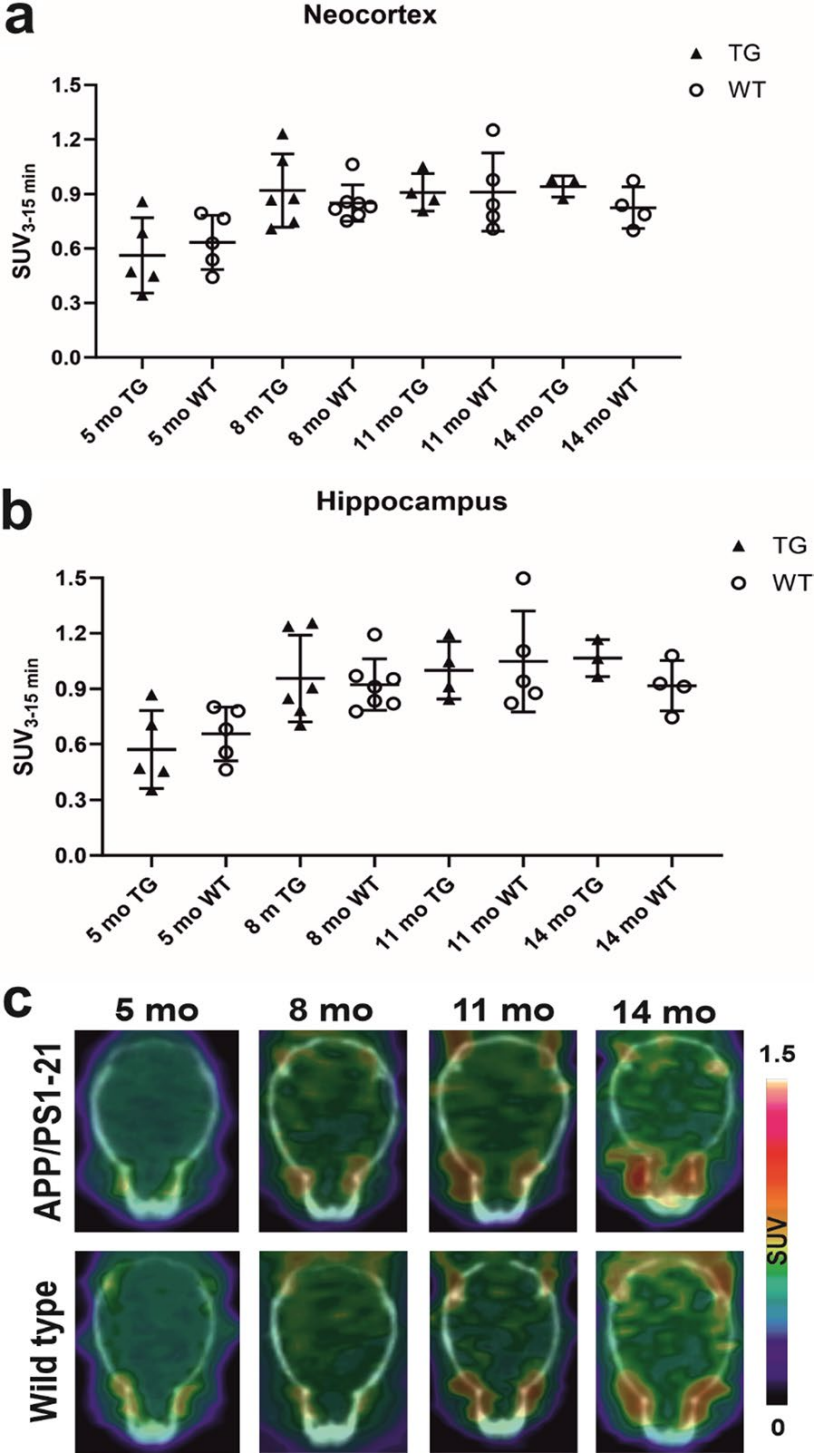
[^11^C]SMW139 longitudinal brain uptake in APP/PS1-21 transgenic (TG) and wild type (WT) mice. **a** and **b** Longitudinal [^11^C]SMW139 standardized uptake values (SUVs) in the neocortex and hippocampus of TG and WT mice at 5, 8, 11 and 14 months of age. Statistical analysis: linear mixed model with compound symmetry covariance structure. **c** Representative axial brain PET/CT images of [^11^C]SMW139 longitudinal SUVs in the same TG and WT mice at 5, 8, 11 and 14 months of age

Longitudinal analysis of [^11^C]SMW139 SUV change in the neocortex and hippocampus revealed no significant difference with ageing from baseline at 5 months to the three follow-up scans at 8, 11, and 14 months (neocortex *p* = 0.53, hippocampus *p* = 0.54) in TG (*n* = 6, including 4 females) or WT (*n* = 7, including 6 females) mice. In addition, we found no significant difference in [^11^C]SMW139 SUVs between TG and age-matched WT mice at any time point (*p* > 0.53 for all time points) in the neocortex or hippocampus (Fig. 2a, b). Representative PET images of longitudinal [^11^C]SMW139 SUVs in the brain of TG and WT mice are shown in Fig. 2c.

**Fig. 3:**
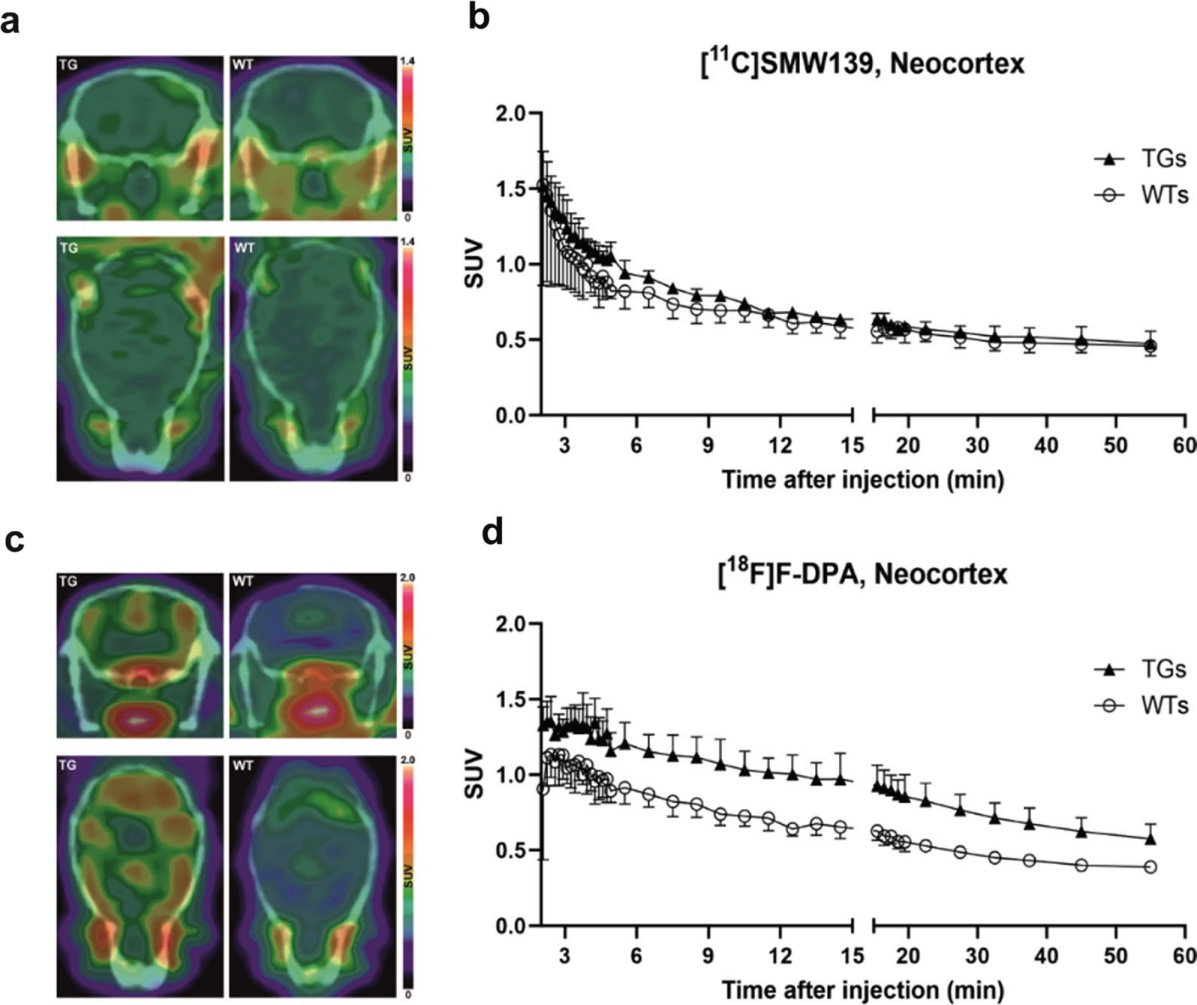
In vivo PET imaging using [^11^C]SMW139 and [^18^F]F-DPA in the same APP/PS1-21 transgenic (TG) and age-matched wild type (WT) mice at 14 months. **a** and **c** Representative coronal and axial brain PET/CT images of TG (*n* = 1) and WT (*n* = 1) mice at 14 months imaged with [^11^C]SMW139 (**a**) and [^18^F]F-DPA (**c**). [^11^C]SMW139 images were summed over 3–15 min and adjusted to the same color scale. [^18^F]F-DPA were summed over 25–50 min and scaled to the same color scale. **b** and **d** Averaged [^11^C]SMW139 (**b**) and [^18^F]F-DPA (**d**) time-activity curves in the neocortex of the same TG (*n* = 3) and WT (*n* = 4) mice at 14 months. Error bars indicate standard deviation

**Fig. 4:**
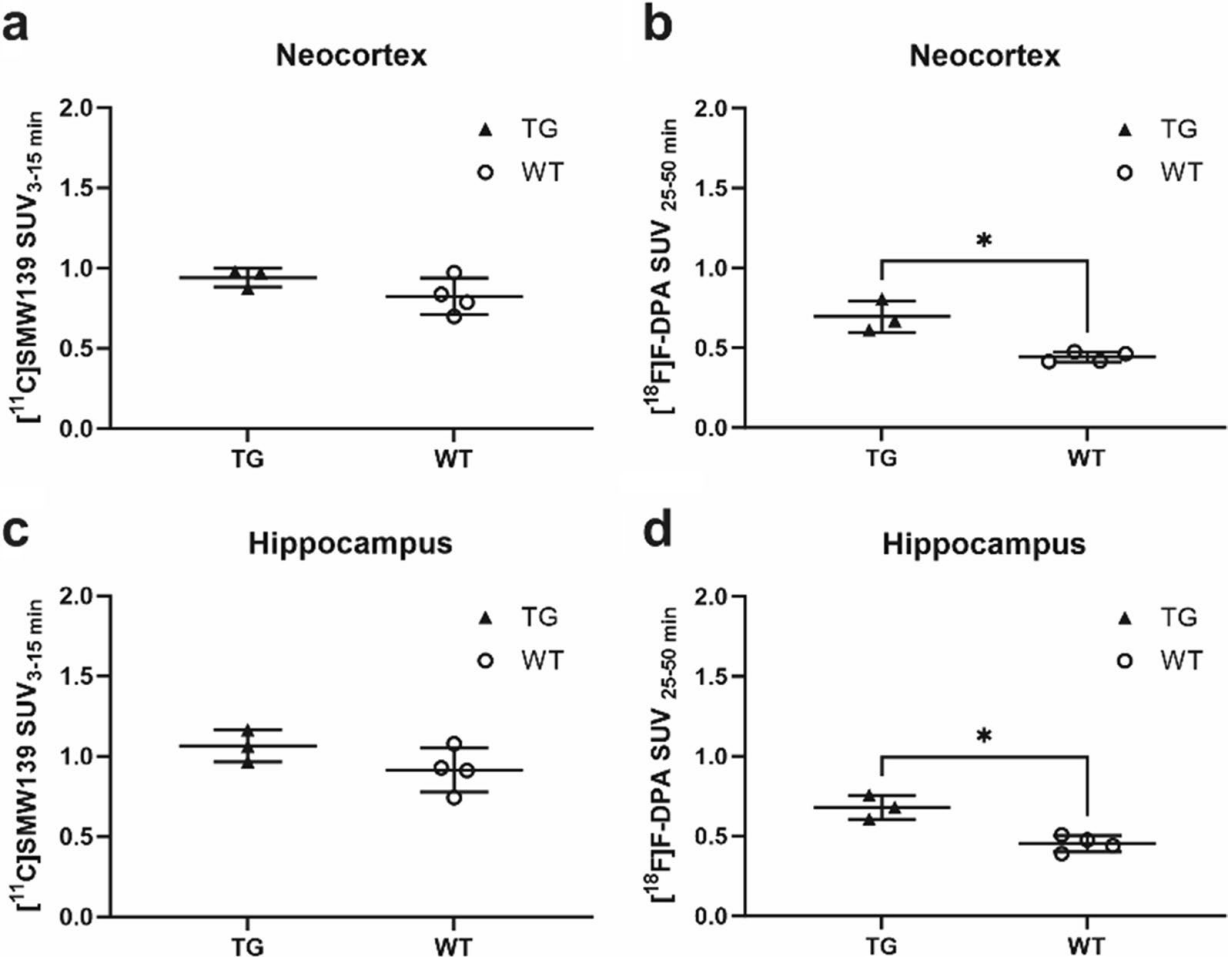
[^11^C]SMW139 and [^18^F]F-DPA standardized uptake value (SUV) in the same APP/PS1-21 transgenic (TG) and wild type (WT) mice at 14 months. **a** and **c** [^11^C]SMW139 averaged SUV in the neocortex (**a**) and hippocampus (**c**) of TG (*n* = 3) and WT (*n* = 4) mice. **b** and **d** [^18^F]F-DPA averaged SUV in the neocortex (**b**) and hippocampus (**d**) of the same TG and WT mice. Statistical test: t-test with Welch’s correction. SUV values are presented with mean and standard deviation. *p < 0.05

At 14 months, mice were imaged with both [^11^C]SMW139 and [^18^F]F-DPA to compare the brain uptake of both radiotracers in the same mice, when neuroinflammation is known to be present in the APPPS1-21 mouse model [29]. [^11^C]SMW139 PET images and averaged time-activity curves in the neocortex showed no difference in radioactivity signal between TG (*n* = 3) and WT (*n* = 4) mice (Fig. 3a, b). On the contrary, higher [^18^F]F-DPA uptake was detected in the PET images and averaged time-activity curves of TG compared to WT mice in the neocortex (Fig. 3c, d). Moreover, [^11^C]SMW139 binding SUVs of TG and WT mice were similar in the neocortex (average SUV 0.94 (0.06) for TGs and 0.82 (0.11) for WTs, *p* = 0.14) and hippocampus (average SUV 1.07 (0.1) for TGs and 0.91 (0.17) for WTs, *p* = 0.15) (Fig. 4a, c). In contrast, in the same animals, significantly higher [^18^F]F-DPA binding SUVs were present in TG compared to WT mice in the neocortex (average SUV 0.7 (0.06) for TGs and 0.44 (0.4) for WTs, *p* = 0.03) and hippocampus (average SUV 0.68 (0.06) for TGs and 0.45 (0.06) for WTs, *p* = 0.01) (Fig. 4b, d).

### Ex vivo brain autoradiography

**Fig. 5:**
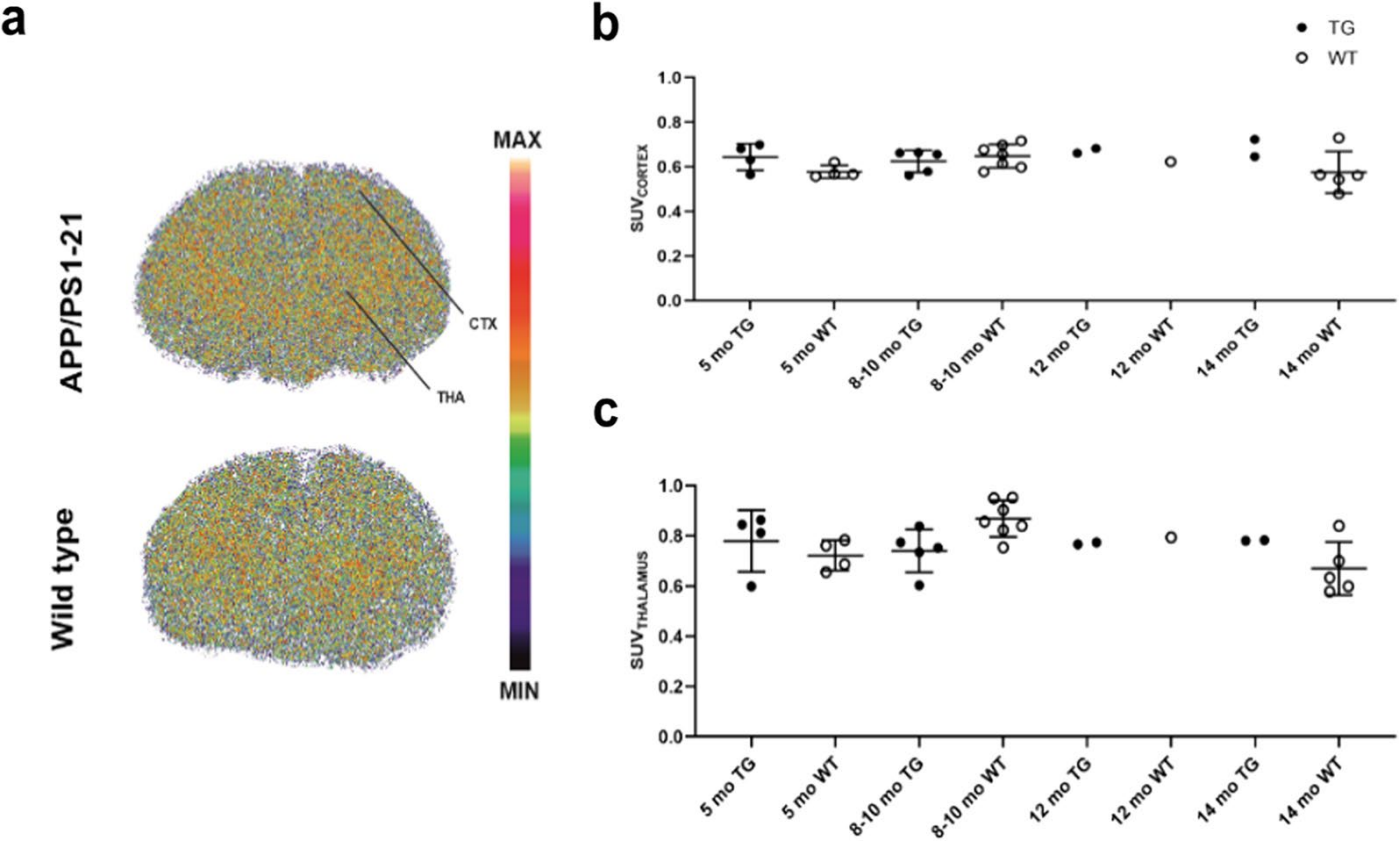
Ex vivo brain autoradiography imaging using [^11^C]SMW139 in APP/PS1-21 transgenic (TG) and wild type (WT) mice. **a** Representative autoradiography images of TG (*n* = 1) and WT (*n* = 1) mice at 8–10 months. Images are adjusted to the same color bar. **b** and **c** [^11^C]SMW139 standardized uptake value (SUV) in cortex (**b**) and thalamus (**c**) of TG (*n* = 4, 5, 2, 2) and WT (*n* = 4, 7, 1, 5) mice at 5, 8–10, 12 and 14 months

Qualitative and quantitative assessment of brain autoradiography images from 30 mice (*n* = 13 TG, *n* = 17 WT) sacrificed 10 min after [^11^C]SMW139 injection showed that the radiotracer has similar uptake in the cortex and thalamus of TG and WT mice with a consistent trend at 5, 8–10, 12 and 14 months (Fig. 5).

### Immunohistochemical staining

**Fig. 6:**
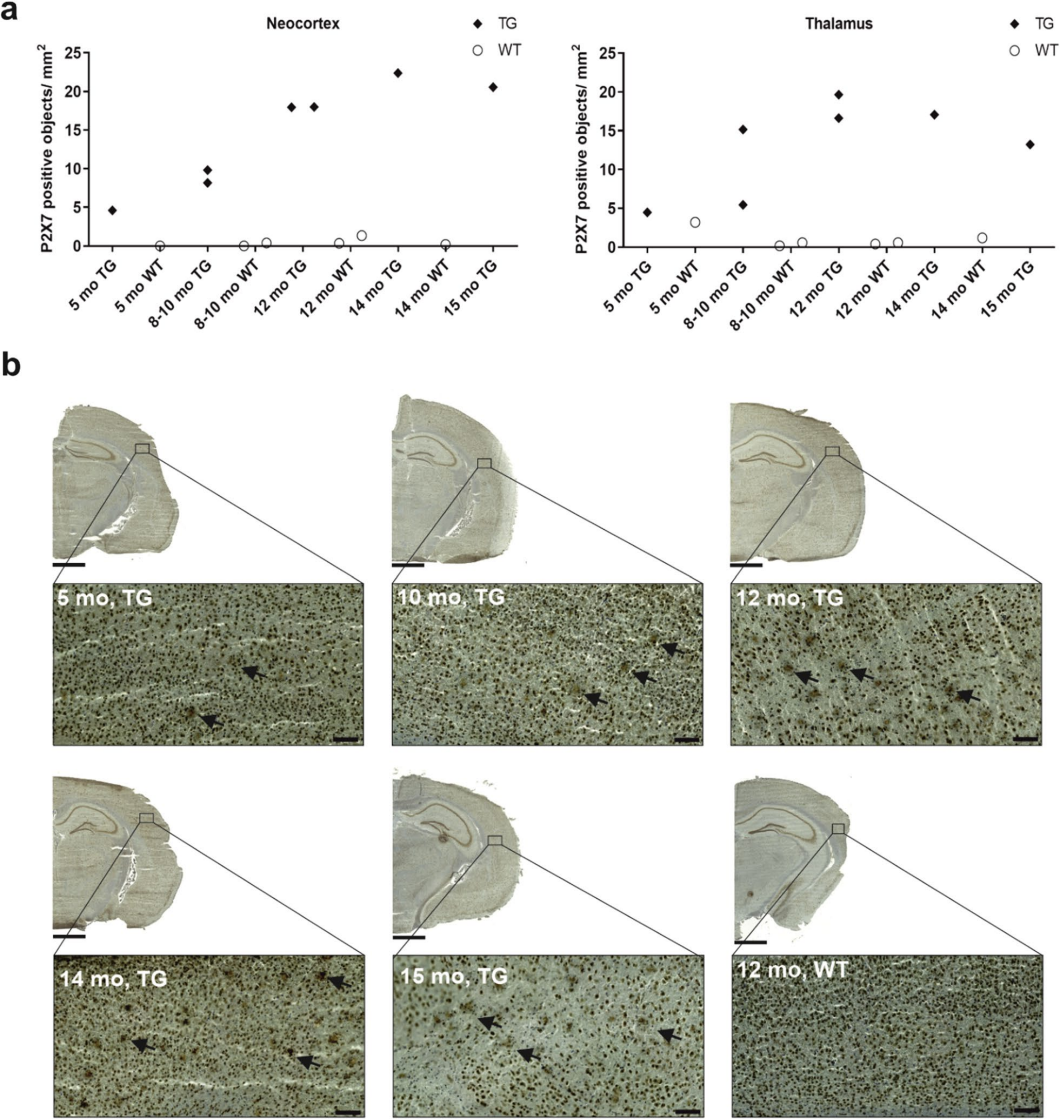
Immunohistochemical staining of P2X7 receptor in brain cryosections of APP/PS1-21 transgenic (TG) and wild type (WT) mice. **a** Quantification of P2X7-positive staining as object counts/mm^2^. Staining was evaluated in the cortex and thalamus of TG and WT mice at 5, 8–10, 12, 14, and 15 months. **b** Representative images of P2X7-positive staining in TG and WT mice at the investigated time points. Arrows point at the P2X7-positive staining detected by the artificial intelligence object detection algorithm. Scale bar = 1000 µm for the half-hemisphere brain section image, 100 µm for the cortex view image. Magnification = 1.5× for the half-hemisphere brain section image, 15.0 × for the cortex view image

Qualitative assessment of P2X7 receptor immunohistochemical staining in mouse brain sections showed a subtle increase in receptor expression in TG mice aged between 5 and 10 months. Expression became slightly more prominent between 10 and 15 months, mainly in the cortex and thalamus. In WT mice, receptor expression remained consistent with ageing. Inline with these observations, quantification of P2X7-positive staining in cortex and thalamus of TG, but not WT, mice demonstrated a subtle increase in receptor expression with ageing (Fig. 6). P2X7-positive staining was also detectable in the white matter corpus callosum of TG, but not WT, mice starting from 8 months (Additional file 1: Fig. S2).

**Fig. 7:**
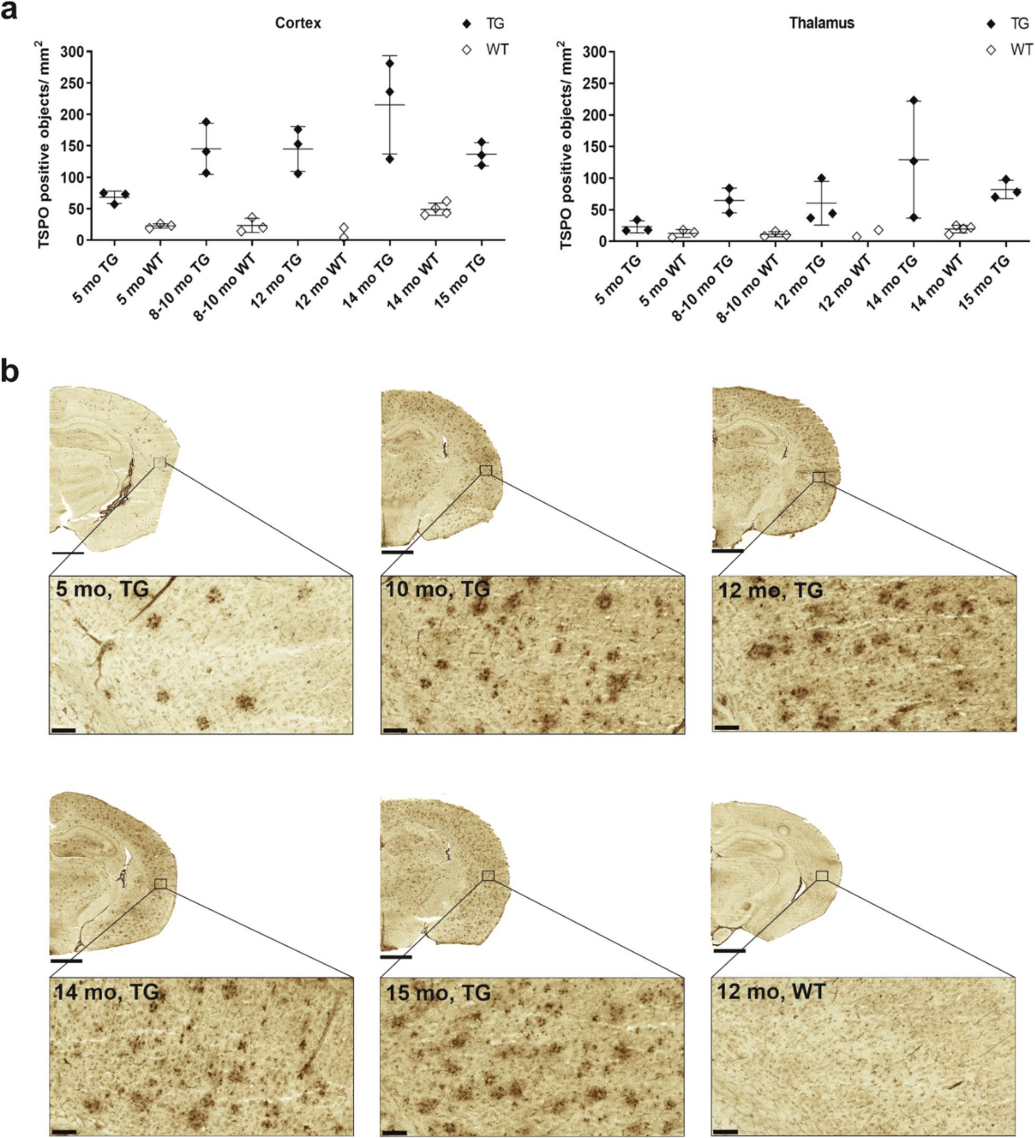
Immunohistochemical staining of TSPO in brain cryosections of APP/PS1-21 transgenic (TG) and wild type (WT) mice. **a** Quantification of TSPO-positive staining as object counts/mm^2^. Staining was evaluated in the cortex and thalamus of TG and WT mice at 5, 8–10, 12, 14, and 15 months. Error bars indicate standard deviation. **b** Representative images of TSPO-positive staining in TG and WT mice at the investigated time points. Scale bar = 1000 µm for the half-hemisphere brain section image, 100 µm for the cortex view image. Magnification = 1.5× for the half-hemisphere brain section image, 15.0 × for the cortex view image

TSPO-positive staining in brain sections of TG mice was detectable already at 5 months. At 8 months, TSPO-positive staining covered the entire brain and further increased in quantity and intensity with ageing. A similar expression pattern was absent in age-matched WT mice. Quantification of TSPO-positive staining in the cortex and thalamus showed that TSPO expression is higher in TG mice and generally increases with ageing, but reaching a plateau at 10 months (Fig. 7). Similar to TSPO, P2Y12-positive staining was detectable in most brain regions of TG, but not WT, mice at 5 months and increased with ageing in all brain regions. Quantification of P2Y12-positive staining in the cortex and thalamus showed a similar increasing trend as TSPO (Additional file 1: Fig. S3).

### Radiometabolite analysis

**Fig. 8:**
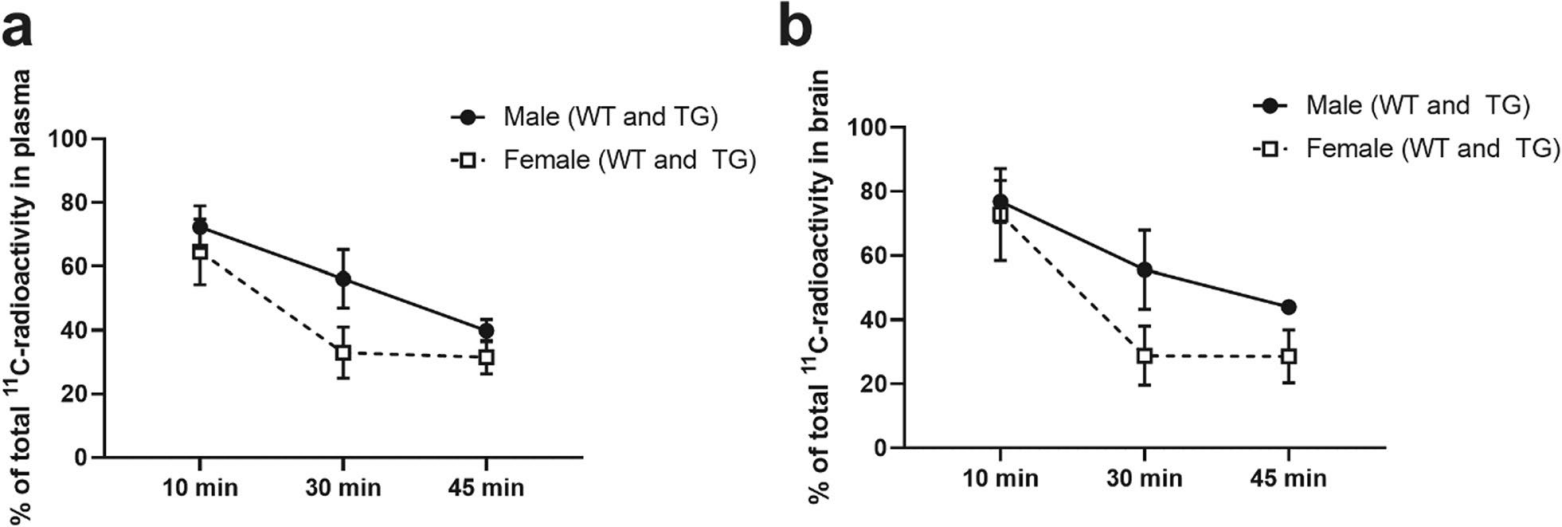
Fractions of unmetabolised [^11^C]SMW139 in APP/PS1-21 transgenic (TG) and wild type (WT) mice plasma and brain homogenates at 10, 30, and 45 min. **a** and **b** Percentage of unmetabolised [^11^C]SMW139 over all radioactivity in male and female mice in plasma (**a**) and brain homogenates (**b**). Error bars indicate standard deviation

The percentage of unmetabolized [^11^C]SMW139 from the total ^11^C-radioactivity in plasma and brain homogenates at 10, 30, and 45 min showed that [^11^C]SMW139 is metabolized quickly. Thirty minutes after radiotracer injection, the mean percentage of [^11^C]SMW139 parent fraction was 33% in plasma and 29% in brain homogenates from female mice, whereas in male mice the percentage was 56% in both plasma and brain homogenates (Fig. 8). The fast metabolism of [^11^C]SMW139 is also accompanied by a gender difference, as the radiotracer is metabolized faster and to a greater extent in female than male WT mice, as seen at 30 and 45 min in plasma and brain homogenates. In the same sex, the radiometabolites of [^11^C]SMW139 accumulate in plasma and brain homogenates in similar fractions and to a similar extent (Fig. 8). At 10, 30, and 45 min, we found no difference between TG and WT mice or mice from different age groups in the percentage of unmetabolized [^11^C]SMW139 in plasma or brain homogenate (t*-*test with Welch’s correction, *p* > 0.25 for all).

## Discussion

Glial reactivity increases with ageing in the APP/PS1-21 mouse model [29], and previous PET studies have demonstrated this phenomenon using different radiotracers [26, 30]. Here, we investigated the P2X7 receptor targeting PET radiotracer, [^11^C]SMW139, for detecting increased glial activity in this mouse model in vivo. In our study, both longitudinal in vivo PET and ex vivo autoradiography imaging of APP/PS1-21 TG and WT mice using [^11^C]SMW139 showed that (1) there were no significant differences in [^11^C]SMW139 uptake between the genotypes in any of the evaluated time points, and (2) brain [^11^C]SMW139 uptake in both TG and WT mice remained around the same level until 14 months. One explanation for these findings is the inadequate expression of P2X7 receptor in the APP/PS1-21 mouse model as presented in this study (discussed further below). Another explanation is the difference between the rodent and human P2X7 receptors, which results in a lower affinity of [^11^C]SMW139 for the rodent compared to the human version of the receptor, complicating the preclinical use of [^11^C]SMW139 [24, 25]. Our findings raise concerns about the ability of other PET radiotracer to quantify P2X7 receptor expression in the APP/PS1-21 mouse model, due to the inadequate expression of the receptor. Moreover, our findings suggest that the APP/PS1-21 mouse model might not be suitable for P2X7 receptor imaging using PET. Janssen et al. reported a ten-fold higher binding of [^11^C]SMW139 in rat striatum overexpressing the human P2X7 receptor compared to the control striatum at one time point [23]. This finding, when compared with our longitudinal imaging results in mice, indicates different binding of [^11^C]SMW139 to the human and mouse P2X7 receptor, and possibly different tracer binding in mice and rats. Additional preclinical imaging studies in rodents would be needed to assess [^11^C]SMW139 binding to both human and rodent P2X7 receptors. Janssen et al. also demonstrated that [^11^C]SMW139 binding did not differ significantly between AD patients and age-matched healthy subjects on post-mortem brain sections, despite a significant increase in P2X7 receptor expression in AD patients [23]. To establish the utility of [^11^C]SMW139 in AD, future studies focusing on evaluating its in vivo binding to the P2X7 receptor in AD patients are needed.

Our immunohistochemical staining findings revealed only a subtle increase in P2X7 receptor expression in the cortex and thalamus of TG mice. In the case of TSPO and P2Y12 receptor, expression in TG mice increased prominently with ageing and compared to age-matched WT mice. At the age of 10 months, expression of all three neuroinflammatory markers in the APP/PS1-21 mouse brain seemed to reach a plateau. This phenomenon aligns with previous findings in this mouse model, as previous reports referred to plateau and variability in detecting microglial activation [37] and AD pathology [28]. A study using brain tissue from both rats and MS patients has indicated a predominant upregulation of P2X7 receptor expression in pro-inflammatory reactive microglia, while P2Y12 receptor expression is mainly upregulated in anti-inflammatory reactive microglia [38]. This same study demonstrated that enhanced expression of microglial P2X7 receptor is accompanied by decreased expression of P2Y12 receptor on the same cells [38]. While further studies in AD are essential to validate these findings, it is conceivable that in our study, the weaker upregulated expression of P2X7 receptor compared to P2Y12 receptor in the APP/PS1-21 mouse model may be attributed to the predominantly anti-inflammatory microglial activation status. This association could be linked to the increased expression of P2Y12 receptor. Nevertheless, additional studies are warranted to characterize the microglial activation status, validate the presence of anti-inflammatory markers in the APP/PS1-21 mouse model, and provide further support for this hypothesis. It is noteworthy that the expression of P2Y12 receptor in AD rodent models is inconsistent. While tau pathology mouse models show reduced expression of P2Y12 receptor, amyloid pathology mouse models show either an increased or an unchanged expression of P2Y12 compared to wild type mice [16]. In our study, we observed a significant upregulation of P2Y12 receptor expression in the APP/PS1-21 mouse model compared to wild-type mice, which is similar to the findings reported in the APP23 mouse model of Aβ deposition [16]. The biological relevance of the inconsistent P2Y12 receptor expression in mouse models is debatable, considering the discrepancy with the downregulated receptor expression in human AD and tauopathies [16]. Nonetheless, the upregulated P2Y12 receptor expression in the APP/PS1-21 mouse model could still be beneficial for imaging purposes, especially in the development of PET tracers for imaging P2Y12 receptor.

In a previous study, we showed that [^18^F]F-DPA targeting TSPO is capable of revealing neuroinflammation trend and its peak at 12–15 months in the APP/PS1-21 mouse model with in vivo PET and ex vivo autoradiography [30]. In the present study, [^18^F]F-DPA showed superiority over [^11^C]SMW139 to detect reactive glia in the APP/PS1-21 mouse model at 14 months. For years, targeting TSPO has been the gold standard for imaging glial activity and neuroinflammation, despite its limitations [17, 18]. Besides, more recent studies highlighted that TSPO expression in humans is related to different phenomena than in mice, and that TSPO-PET signals in humans reflect the density of inflammatory cells (microglia and macrophages) rather than the activation status in mice, raising concerns about the translatability of TSPO-PET from rodents to humans [39–41]. Finding a superior imaging marker to TSPO remains a hurdle, although several PET ligands targeting other imaging markers of neuroinflammation are being explored [17]. One challenge is the lack of glia selectivity; like TSPO, the majority of the potential imaging markers are expressed in different glial cell types, which react heterogeneously during different phases of neuroinflammation, resulting in unpredictability of the imaging markers expression profiles, thus complicating the in vivo PET imaging quantification. An imaging marker superior to TSPO needs to be specific for a certain glial cell type, and have different expression patterns in physiological and neuroinflammatory conditions.

Defining the time frame for quantifying [^11^C]SMW139 in vivo uptake was complicated by the fast metabolic profile in mice, and the presence of radioactive metabolites in the brain tissue. In a previous study with [^11^C]SMW139, we detected up to three brain penetrant radiometabolites of [^11^C]SMW139 in mouse brain homogenates and one radiometabolite more in plasma already at 10 min after tracer injection [32]; similar findings were also reported by Brumberg and colleagues [42]. Accordingly, an appropriate time frame needs to be selected in which there is still a reasonable percentage of the parent fraction and as little interference as possible from the radiometabolite fraction. Considering that [^11^C]SMW139 started to washout around 90 s after injection in mice, 3–15 min was selected to calculate the averaged SUVs for group comparisons. Although it is not known if these three radiometabolites of [^11^C]SMW139 bind to P2X7 receptor or another target, their presence in the brain disturbs the PET data quantification and hamper the utilization of [^11^C]SMW139 in any in vivo PET application. Defining the time frame for quantifying [^18^F]F-DPA in vivo uptake in the same mice was more straightforward. The time frame 25–50 min was used to calculate the averaged SUVs, considering that [^18^F]F-DPA has no brain penetrating radiometabolites in mice [30]. SUVs were used to analyze both tracers brain uptake, as a reference region for binding ratio calculations was not found. In plasma samples and brain homogenates, [^11^C]SMW139 parent fraction was similar in mice at all ages, indicating that [^11^C]SMW139 metabolism is independent of aging. However, our results show that the [^11^C]SMW139 parent fraction decreased faster in plasma and brain homogenates from female compared to male mice, indicating a gender difference in [^11^C]SMW139 metabolism in mice. In contrast, no difference has been reported in [^11^C]SMW139 plasma metabolism between male and female rats [22]. Moreover, our results showed that the [^11^C]SMW139 parent fraction at 45 min was similar in plasma and brain homogenate (40% and 44%, respectively), whereas the [^11^C]SMW139 parent fraction at 45 min in male rats was different in plasma (42%) and brain homogenates (66%) [22], and fractions within the same range were reported in female rats at the same time points [24]. Taken together, these findings show that [^11^C]SMW139 is metabolized to a greater extent in mouse brain (44%) than rat brain (66%).

There were strengths in this study. First, using a longitudinal imaging design with [^11^C]SMW139 allowed the assessment of reactive microglia in the same animals with ageing. The investigated groups, comprising 6 TG and 7 WT mice, were small because of the longitudinal nature of the study. This design involved repeated imaging of the same animals, which enables making within-subject comparisons of imaging findings, controlling for between-subject variability. This is an excellent advantage of in vivo PET imaging, particularly when assessing age-related pathophysiological changes. Therefore, despite the smaller number of mice, the results remain reliable because each mouse served as its own control. Second, performing the radiotracers comparison in the same animals excludes potential variability due to using different animals. Third, the availability of immunohistochemical staining of both radiotracers imaging targets assessed in explaining the in vivo imaging findings. On the other hand, our study had limitations. Firstly, it was not possible to scan each mouse of the starting cohort at all four follow-up time points because some mice needed to be sacrificed during the study. Secondly, both male and female mice were included in this imaging study. Ideally, including only one gender would help mitigate potential variability arising from gender differences. Thirdly, fewer animals per age group were used in the immunohistochemical staining of P2X7 receptor in comparison to TSPO and P2Y12 receptor. Additionally, immunohistochemical staining of P2X7 receptor and TSPO were performed in the same animals only at two of the four investigated time points, which did not allow for a thorough comparison of the imaging markers solely based on the immunohistochemical staining findings. Moreover, due to the inferior quality and different features of P2X7 receptor staining compared to that of TSPO and P2Y12 receptor, we were forced to use a different analysis method to quantify P2X7 receptor staining, which could be seen as a limitation to the direct comparison of imaging markers. Lastly, neither P2X7 receptor nor TSPO are expressed exclusively in microglia, rather generally in glia and the potentially infiltrating macrophages to the central nervous system from the periphery, thus [^11^C]SMW139 and [^18^F]F-DPA in vivo brain uptake represent binding to glia collectively, not solely microglia.

## Conclusions

This study aimed to use the P2X7 receptor as an imaging marker for reactive glia in an Alzheimer's disease mouse model. However, [^11^C]SMW139, which has lower affinity for the rodent P2X7 receptor compared to the human version of the receptor, was unable to measure the low expression of P2X7 receptor in the APP/PS1-21 mouse model. Additionally, the fast metabolism of [^11^C]SMW139 in mice and the presence of several brain-penetrating radiometabolites significantly impacted the analysis of in vivo PET signal of the tracer. Finally, [^18^F]F-DPA targeting TSPO was more suitable for imaging reactive glia and neuroinflammatory processes in the APP/PS1-21 mouse model, based on the findings presented in this study and previous studies with this mouse model.

### Supplementary Information

**Additional file 1.** Supplementary methods.

## Data Availability

The datasets used and/or analyzed during the current study are available from the corresponding author on reasonable request.

## Abbreviations

AD: Alzheimer’s disease
Aβ: Beta-amyloid
P2X7: Purinergic 2 type X receptor subtype 7
TSPO: Translocator protein-18 kDa
P2Y12: Purinergic 2 type Y receptor subtype 12
PET: Positron emission tomography
MS: Multiple sclerosis
TG: Transgenic
WT: Wild type
MRI: Magnetic resonance imaging
VOI: Volume of interest
SUV: Standardized uptake value
