# Correction: Glial reactivity in a mouse model of beta-amyloid deposition assessed by PET imaging of P2X7 receptor and TSPO using [^11^C]SMW139 and [^18^F]F-DPA

**DOI:** 10.1186/s13550-024-01103-8

**Published:** 2024-05-03

**Authors:** Obada M. Alzghool, Richard Aarnio, Jatta S. Helin, Saara Wahlroos, Thomas Keller, Markus Matilainen, Junel Solis, Jonathan J. Danon, Michael Kassiou, Anniina Snellman, Olof Solin, Juha O. Rinne, Merja Haaparanta‑Solin

**Affiliations:** 1 PET Preclinical Imaging Laboratory, Turku PET Centre, University of Turku, Tykistökatu 6 A, Turku, 20520 Finland; 2 Medicity Research Laboratory, University of Turku, Tykistökatu 6 A, Turku, 20520 Finland; 3 Drug Research Doctoral Programme, University of Turku, Turku, Finland; 4 Turku University Hospital, Turku PET Centre, Kiinamyllynkatu 4-8, 20520 Turku, Finland; 5 Radiopharmaceutical Chemistry Laboratory, Turku PET Centre, University of Turku, Kiinamyllynkatu 4-8, FI-20520 Turku, Finland; 6 Turku BioImaging, Åbo Akademi University and University of Turku, Turku, Finland; 7 School of Chemistry, The University of Sydney, Sydney, NSW 2006 Australia; 8 Department of Chemistry, University of Turku, Henrikinkatu 2, Turku, 20500 Finland; 9 Accelerator Laboratory, Turku PET Centre, Åbo Akademi University, Kiinamyllynkatu, Turku, 4‑8, 20520 Finland; 10 Department of Neurology, Turku University Hospital, Kiinamyllynkatu 4-8, 20520 Turku, Finland

**Correction: EJNMMI Research (2024) 14:25**

10.1186/s13550-024-01085-7.

Following publication of the article, the following errors were brought to the attention of the journal: In Figures 5, 6, and 7, white squares had been erroneously included behind the brain section images during production of the article, and the affiliations information of the article was incomplete. The published article has since been corrected.

