# Supplementary material for: Glial reactivity in a mouse model of beta-amyloid deposition assessed by PET imaging of P2X7 receptor and TSPO using [11C]SMW139 and [18F]F-DPA
Source: EJNMMI Res. 2024 Mar 6;14:25. doi: 10.1186/s13550-024-01085-7 (PMC10917722; doi:10.1186/s13550-024-01085-7)
Supplement: Supplementary file 1 — Additional file 1. Supplementary methods. [file 13550_2024_1085_MOESM1_ESM.docx]

# Glial reactivity in a mouse model of beta-amyloid deposition assessed by PET imaging of P2X7 receptor and TSPO using [^11^C]SMW139 and [^18^F]F-DPA

Obada M. Alzghool^1,2,3*^, Richard Aarnio^1,2,3^, Jatta S. Helin^1,2^, Saara Wahlroos^1^, Thomas Keller^1^, Markus Matilainen^1^, Junel Solis^4^, Jonathan J. Danon^5^, Michael Kassiou^5^, Anniina Snellman^1^, Olof Solin^1,6,7^, Juha O. Rinne^1^ and Merja Haaparanta-Solin^1,2^

^1^Turku PET Centre, University of Turku, Kiinamyllynkatu 4-8, FI-20520 Turku, Finland

^2^Medicity Research Laboratory, University of Turku, Tykistökatu 6 A, FI-20520 Turku, Finland

^3^Drug Research Doctoral Programme, University of Turku, Turku, Finland

^4^Turku BioImaging, Åbo Akademi University and University of Turku, Turku, Finland

^5^School of Chemistry, The University of Sydney, Sydney, NSW 2006, Australia

^6^Department of Chemistry, University of Turku, Henrikinkatu 2, FI-20500 Turku, Finland

^7^Accelerator Laboratory, Turku PET Centre, Åbo Akademi University, Kiinamyllynkatu 4-8, FI-20520 Turku, Finland

Corresponding author: Obada M. Alzghool, Turku PET Centre, University of Turku, Tykistökatu 6 A, 4th floor, FI-20520, Turku, Finland, [obalzg@utu.fi](mailto:obalzg@utu.fi)

# Supplementary methods

## Immunohistochemical staining

**Table S1.** Immunohistochemical staining protocol for P2X7, TSPO and P2Y12 receptors using 20-µm fresh-frozen non-fixed mouse brain sections. The washing buffer was Tris-HCl 0.05 M, pH 7.6 (Reagena 112270) with 0.05% Tween 20 detergent. All incubations were done at room temperature (RT).

| **Step** | **Details** | | |
| --- | --- | --- | --- |
| 1. **Preparation** | Let sections dry, 30 min, RT | | |
| 1. **Fixation** | Formalin incubation, 10 min, RT | | |
| 1. **Washing** | Washing buffer, 2x3 min | | |
| 1. **Antigen retrieval** | Citrate buffer (pH 6) incubation, pre-heated to boil, 20 min, RT | | |
| 1. **Washing** | Washing buffer, 2x3 min | | |
| 1. **Endogenous hydrogen peroxidase enzyme blocking** | 0.3% H_2_O_2_ in buffer, 10 min, RT | | |
| 1. **Rinsing** | Rinse slides in dH_2_O | | |
| 1. **Pre-protein blocking** | Normal antibody diluent (WellMed BD09-125) incubation, 10 min, RT | | |
| 1. **Primary antibody incubation in buffer** | Anti-P2X7  (APR-008)  1:1000  60 min | Anti-P2Y12  (AS-55043A)  1:1000  60 min | Anti-PBR  (EPR5384)  1:3000  60 min |
| 1. **Rinsing** | Rinse slides in dH_2_O | | |
| 1. **Secondary antibody incubation** | Goat anti-rabbit IgG HRP (WellMed DPVR110HRP), 30 min, RT | | |
| 1. **Rinsing** | Rinse slides in dH_2_O | | |
| 1. **Substrate** | Substrate DAB (WellMed BS04-110) incubation, 10 min, RT | | |
| 1. **Rinsing** | Rinse slides in dH_2_O | | |
| 1. **Counter stain (if needed)** | Mayers hematoxylin (histolab 01820), 1 min | | |
| 1. **Rinsing** | Rinse slides in dH_2_O | | |
| 1. **Dehydration and mounting** | Alcohol series and mounting medium | | |

## **Quantification of P2X7 receptor staining using the artificial intelligence object detection algorithm You Only Learn One Representation (YOLOR)** (1)

A dataset with a total of 1,765 images (each 800 x 800 pixels) was annotated for quantification, with a random split of 60%-20%-20%. This dataset was used to train a YOLOR model to detect P2X7-positive staining. Training was done using the PyTorch framework with an NVIDIA GeForce RTX 3070 graphics processing unit, batch size 20, input image sizes of 448 x 448 pixels, for 200 epochs. The trained model was used to identify spots of PX27-positive staining in the NC and THA images from both WT and TG animals. Python scripting was used to measure the ratio of P2X7-positive objects count to NC or THA area.

**Source code for quantification of P2X7-positive staining:** The image processing source code for the quantification of P2X7-positive staining is available via the following link: <https://github.com/Turku-BioImaging/idt-p2x7-object-detection>

## Radiometabolite analysis

The radiometabolite analysis was initially performed using high-performance thin-layer chromatography (HPTLC), but the method was further improved during the study and later analysis performed using thin-layer chromatography (TLC). During the transition stage of the method change, the samples were run using both methods to validate that the parent fraction results were the same.

**High-performance thin-layer chromatography (HPTLC):** The obtained plasma was mixed with acetonitrile (plasma:acetonitrile 1:1.4 *v/v*) in an Eppendorf tube to precipitate the plasma proteins. The mixture was vortexed and centrifuged (12100 ×g, 90 s) to separate the protein-free supernatant, and 5-12 µL aliquots of the supernatant were placed on the HPTLC plate (HPTLC, silica gel 60 RP-18, art no. 1.05914.0001, Merck KGaA, Darmstadt, Germany).

A piece of mouse brain was cut and placed into a glass homogenizer and homogenized into the mobile phase. The volume of mobile phase used as solvent was kept as small as possible. The homogenate was centrifuged and aliquots of 8-20 µL were applied onto the same HPTLC plate as the standard and plasma. The application line was 1.5 cm from the bottom of the plate, and the migration distance was 4 cm. The plate was dried carefully on low heat and placed in an elution chamber (10 × 10 cm Twin Trough Chamber, Camag, Muttenz, Switzerland; 75:25:0.1 acetonitrile:water:trifluoroacetic acid (*v/v/v*) as the mobile phase; volume of mobile phase: 5 mL in each compartment). The migration time was 35-50 min.

**Thin-layer chromatography (TLC):** The TLC method was published previously (2). The main differences from the HPTLC method were that TLC (Silica gel 60 RP-18. art no. 1.05559.0001, Merck KGaA, Darmstadt, Germany) plates were used, the mobile phase was 65:35:0.1 acetonitrile:water:trifluoroacetic acid (*v/v/v*), and the plasma:acetonitrile ratio was 1:2 when precipitating the plasma proteins. The amount of plasma and brain samples applied was up to 16 µL. The migration time for 4 cm of travel was 6 min.

Common for both methods:

A radioactive standard of [^11^C]SMW139 was prepared in the mobile phase. The radioactivity concentration was chosen so that it corresponded roughly to the radioactivity concentration of the plasma sample. The standard was placed on each plate on which samples were analyzed.

After the elution, the plates were dried carefully using the medium-heat setting and fixed in an autoradiography developing cassette and exposed to an erased autoradiography imaging plate (BAS-TR2025, Fuji Photo Film Co., Ltd., Tokyo, Japan) for approximately 1 hour. The autoradiography plate was digitalized using a phosphorimager (BAS-5000, Fuji Photo Film Co., Ltd., Tokyo, Japan) and analyzed using Aida Image Analyzer (v.4.22, Elysia-Raytest GmbH, Straubenhardt, Germany).

The parent fraction was analyzed as the photostimulated luminescence corresponding to the unchanged [^11^C]SMW139 (identified by the radioactive standard) divided by the total photostimulated luminescence of the whole sample. Correction for background radioactivity was performed.

References

1. Wang C-Y, Yeh I-H, Liao H-YM. You Only Learn One Representation: Unified Network for Multiple Tasks. 2021 May 10 [cited 2022 Jun 28]; Available from: https://arxiv.org/abs/2105.04206v1

2. Aarnio R, Alzghool OM, Wahlroos S, O’Brien-Brown J, Kassiou M, Solin O, et al. Novel plasma protein binding analysis method for a PET tracer and its radiometabolites: A case study with [11C]SMW139 to explain the high uptake of radiometabolites in mouse brain. J Pharm Biomed Anal. 2022 Sep 20;219:114860.

# Supplementary figures


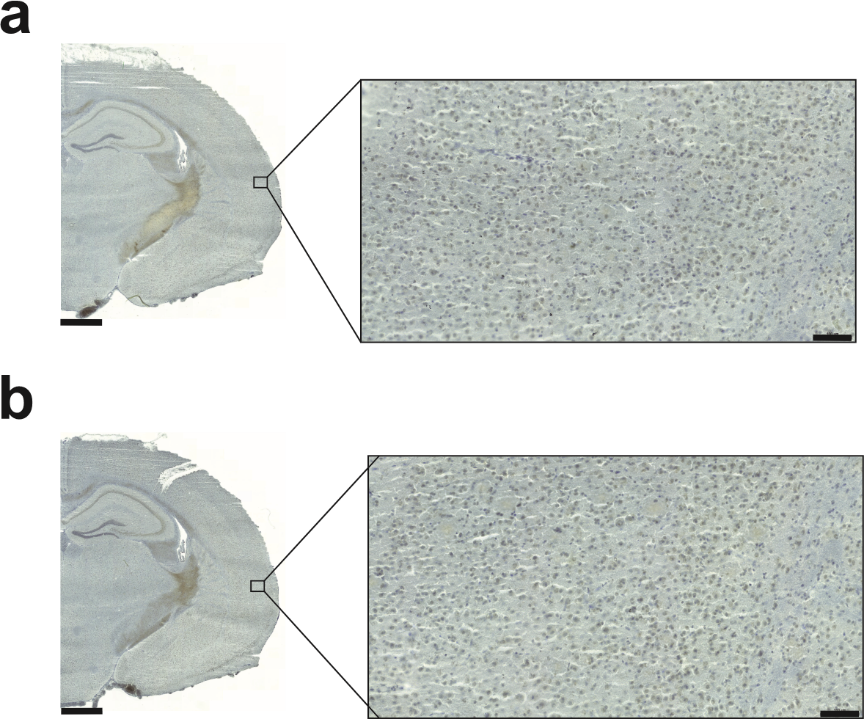


**Fig S1.** Negative staining of P2X7 (A) and TSPO (B) receptors in TG mouse 10 months old by omitting the primary antibody confirms antibody specificity. Scale bar = 1000 µm for the half hemisphere brain section image, 100 µm for the cortex view image. Magnification = 1.5x for the half hemisphere brain section image, 15.0x for the cortex view image.

**
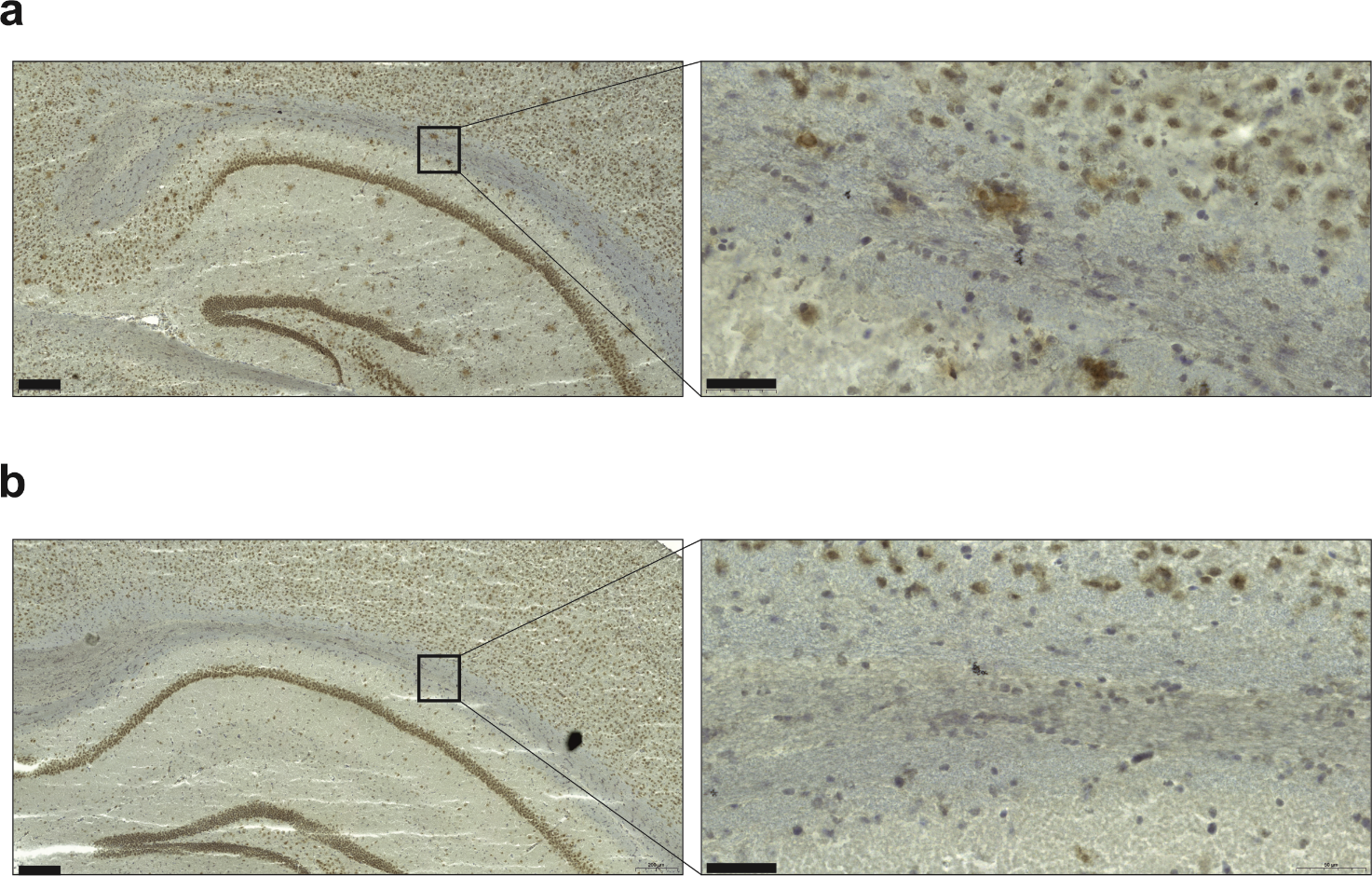
**

**Fig S2.** Representative images of P2X7 receptor positive staining detectable in the white matter corpus callosum of TG (A) but not WT (B) mouse at 14 mo. Scale bar = 200 µm for the half hemisphere brain section image, 50 µm for the corpus callosum view image.

**
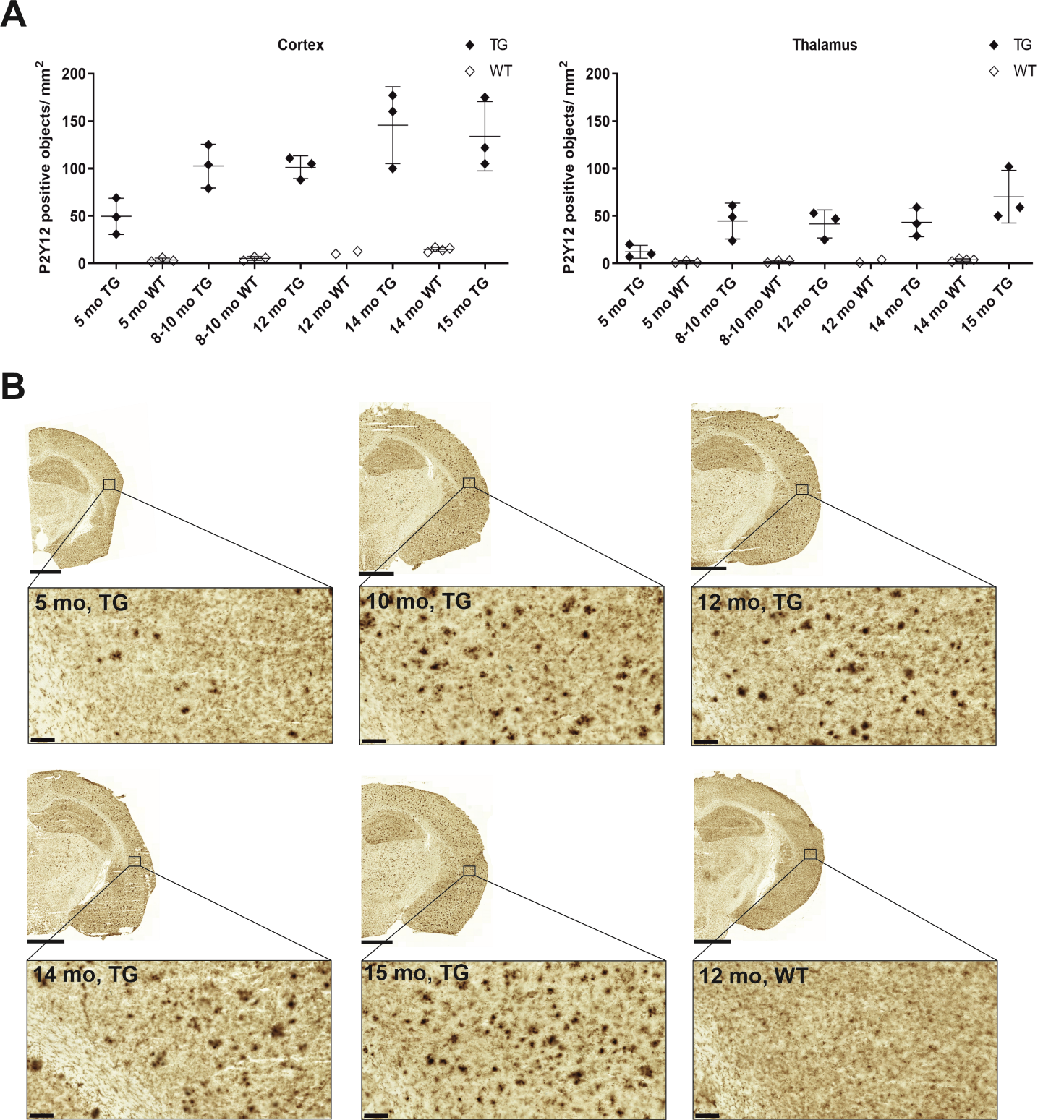
**

**Fig S3.** Immunohistochemical staining of P2Y12 receptor in brain cryosections of APPPS1-21 transgenic (TG) and wild-type (WT) mice. (A) Quantification of P2Y12-positive staining as object counts/mm^2^. Staining was evaluated in the cortex and thalamus of TG and WT mice at 5, 8-10, 12, 14, and 15 months. Error bars indicate standard deviation. (B) Representative images of P2Y12-positive staining in TG mice at 5, 10, 12, 14 and 15 mo, and WT mice at 12 mo. Scale bar = 1000 µm for the half hemisphere brain section image, 100 µm for the cortex view image. Magnification = 1.5x for the half hemisphere brain section image, 15.0x for the cortex view image.
